# Analysis of influenza B virus lineages and the HA1 domain of its hemagglutinin gene in Guangzhou, southern China, during 2016

**DOI:** 10.1186/s12985-018-1085-5

**Published:** 2018-11-14

**Authors:** Feng Ye, Xiao-juan Chen, Wen-da Guan, Si-hua Pan, Zi-feng Yang, Rong-chang Chen

**Affiliations:** State Key Laboratory of Respiratory Disease, National Clinical Research Center for Respiratory Disease; Guangzhou Institute of Respiratory Disease, First Affiliated Hospital of Guangzhou Medical University, Guangzhou Medical University, 151 Yan Jiang Road, Guangzhou, Guangdong 510120 People’s Republic of China

**Keywords:** Antigenic epitope, *HA1* gene, Influenza B virus

## Abstract

**Background:**

Few studies have analyzed influenza B virus lineages based on *hemagglutinin A* (*HA*) gene sequences in southern China. The present study analyzed the *HA* gene and the lineages of influenza B virus isolates from Guangzhou during 2016, and compared our results with the WHO-recommended vaccine strain.

**Methods:**

Ninety patients with influenza B were recruited from the First Hospital of Guangzhou Medical University. Throat swab specimens of 72 patients had high viral loads. Among these 72 isolates, the *HA1* domain of the HA gene in 43 randomly selected isolates was sequenced using reverse transcription-polymerase chain reaction (RT-PCR), and analyzed using MEGA 5.05.

**Results:**

Eight of the 90 patients (8.9%) also had influenza A virus infections. Analysis of the 43 influenza B virus isolates indicated that 34 (79.1%) were from the Victoria lineage and 9 (20.9%) were from the Yamagata lineage. A comparison isolates in our Victoria lineage with the B/Brisbane/60/2008 strain indicated 12 mutation sites in the *HA1* domain, 4 of which (I132V, N144D, C196S, and E198D) were in antigenic epitopes. A comparison of isolates in our Yamagata lineage with the B/Phuket/3073/2013 stain indicated 5 mutation sites in the *HA1* domain, none of which was in an antigenic epitope. None of the isolates had a mutation in regions of the neuraminidase gene (*NA*) that are known to confer resistance to NA inhibitors.

**Conclusion:**

In Guangzhou during 2016, most influenza B virus isolates were from the Victoria lineage, in contrast to the vaccine strain recommended by the WHO for this period.

**Electronic supplementary material:**

The online version of this article (10.1186/s12985-018-1085-5) contains supplementary material, which is available to authorized users.

## Introduction

Influenza B is an acute upper respiratory tract infection that is caused by the influenza B virus, a negative-sense, single-stranded RNA virus in the Orthomyxoviridae family. The influenza B virus was first isolated in the 1940 pandemic in the United States (strain B/Lee/40). Since 1983, influenza B viruses have been classified into two distinct lineages according to their antigenicity and gene sequences: B/Victoria/2/87-like (Victoria) and B/Yamagata/16/88-like (Yamagata) [1, 2]. Gene segment reassortment between and within these lineages has contributed to the genetic diversity of this virus [3].

Research indicates that the Victoria and Yamagata lineages have circulated over time in different geographical regions, including in China [4–12], and that the dominant lineages have changed over time in different geographical locations. For example, during the 1990s, Yamagata strains were more prevalent, particularly in northern China [13, 14], but the Victoria strains were prevalent in China from 2004 to 2008 [5, 15–17].

Along with neuraminidase (NA), hemagglutinin (HA) is an antigenic glycoprotein on the surface of the influenza virus that can also induce the production of neutralizing antibodies. HA is responsible for viral binding to the cell membranes of host cells, and has therefore been used as a key antigen for the development of vaccines [18]. Mutations in *HA* can lead to viral antigenic transformation, and mutations in *NA* are primarily associated with virulence and resistance [19, 20]. Specifically, the influenza B virus may change its HA antigenicity to escape the immune system of the host, causing repeated epidemics in the general population [2]. Thus, analysis of the HA1 domain of *HA* may help to identify strains with new gene mutations, and provide insights that could help to prevent and control influenza.

World Health Organization (WHO) data indicate that the prevalence of influenza B increased significantly in China during 2016, in contrast to previous reports [21]. Furthermore, phylogenetic analysis of the influenza B virus *HA* and *NA* sequences indicated considerable changes in the prevalence of the different lineages in Guangzhou during 2009 to 2010, a finding that has important implications for vaccination policies [9]. This study investigated the HA1 domain of the *HA* gene of influenza B virus isolates from Guangzhou during 2016. We systemically investigated *HA* gene mutation(s) of the influenza B virus and compared the strains that were actually present with the vaccine strain recommended by the WHO for the Northern hemisphere.

## Materials and methods

### Study design

Consecutive patients with influenza B were recruited from the First Hospital of Guangzhou Medical University and nearby communities from January 2016 to May 2016. The inclusion criteria were: *(i)* axillary body temperature of at least 38 °C; *(ii)* diagnosis of influenza according to the 2011 Guideline for the Diagnosis and Treatment of Influenza (2011) [22], with nasal congestion, sore throat, cough, muscle or joint pain, fatigue, and/or headache; and *(iii)* a positive result from the rapid influenza B virus antigen test (Clearview Extract Influenza A&B, Abbott Laboratories, USA), a rapid immunochromatographic assay, or from a quantitative fluorescence RT-PCR assay of a nose/throat swab. Patients were excluded if they or their parents/guardians refused to participate. Informed consent was obtained from each patient or parent/caretaker before enrolment. This study was approved by the Institutional Research Board (IRB) of the First Hospital of Guangzhou Medical University (No: 2014013).

A total of 90 patients with influenza B were recruited, 8 of whom had both influenza A and influenza B. All 72 samples with high viral loads were analyzed. Some throat swab specimens that had low viral loads (Ct value of ~ 38.2, corresponding to 10 copies) were not included because they had negative results in viral cultures using MDCK cells (see below). To detect viral load, a diagnostic kit for influenza A and B virus RNA (PCR-Fluorescence Probing) from Guangzhou Institute of Respiratory Disease Pharmaceutical Technology Co., Ltd. (China) was used. The following PCR primers and probe were used: IFB Forward primer: CGAGCGTYTTAATGAAGGACATTC; IFB Reverse primer: GACCAAATTGGGATAAGACTCCC; Probe: AM-AGCCAATTCGAGCAGCTGAAACTGCG-BHQ1.

### Specimen collection

Throat swab specimens were obtained from inpatients and outpatients who were diagnosed with influenza B at the First Hospital of Guangzhou Medical University during 2016.

### Influenza B virus culture

MDCK cells were seeded into 96-well plates. When the confluence of MDCK cells was 90 to 95%, the medium was removed, and plates were washed with PBS twice. Then, virus-containing medium was added to each well (100 μL/well; 3–4 wells/specimen). The virus culture medium contains MEM supplemented with fetal bovine serum (FBS), trypsin, and penicillin/streptomycin (all from Life Technologies, Carlsbad, CA, USA). The plates were incubated at 37 °C for 48 h. Cytopathic effect (CPE) was detected by visualizing changes in MDCK morphology, including cell rounding, syncytium formation, and appearance of inclusion bodies. The supernatants of specimens with CPE and a positive result in the hemagglutination assay (described below) were stored at − 80 °C.

### Hemagglutination assay

Viral suspensions were added to a V-type 96-well plate (50 μL/well) with 0.5% red blood cells (RBCs) isolated from chicken blood (50 μL/well). After a 30-min incubation at 4 °C, the presence of RBC agglutination was recorded. The absence of agglutination was indicated by settling of RBCs to the bottom of the well.

### Extraction of viral nucleic acids

Total RNA was extracted from 38 virus samples that were randomly selected from the 72 isolates using the Trizol reagent (Life Technologies) according to the manufacturer’s instructions. Only sequencing, not quantitation, was performed. RT-PCR was performed using a QIAGEN OneStep RT-PCR Kit (Hilden, Germany), with SYBR Green I for detection and viral RNA as a template. The primers were as follows: HA1 sense, 5´-TGTAAAACGACGGCCAGTAGCAGAAGCRKWGC-3′ where R = G A (purine), K = G T (keto), and W = A T (weak bonds), and HA1 antisense 5´-AGGAAACAGCTATGACCCTCATCTTCACTGTTTATTATTCC-3′; NA sense, 5´-TGTAAAACGACGGCCAGTAGCAGAAGCAGAGC-3′ and NA antisense 5´-CAGGAAACAGCTATGACCTGTAGTAACAAGAGCATT-3′. Primers were synthesized in Liuhe Huada Gene Biotech Co., Ltd. (Beijing, China). The PCR procedure was: pre-denaturation at 50 °C for 15 min and 94 °C for 2 min; 35 cycles of denaturation at 94 °C for 30 s, annealing at 50 °C for 90 s and extension at 72 °C for 1 min, and a final extension at 72 °C for 10 min.

### Sequencing and analysis

The purified RT-PCR products were sequenced at the Liuhe Huada Gene Biotech Co., Ltd., analyzed using the SeqMan mode of DNAStar software, and assembled using Sequencher software. The assembled sequences were compared with sequences of vaccine strains published by the WHO in NCBI using the Molecular Evolutionary Genetics Analysis version 5.05 (MEGA 5.05) software. The open reading frame (ORF) was reserved, and the noncoding region was removed. The Neighbor-Joining method was used to determine a phylogenetic tree (cladogram) based on the HA1 domain of the *HA* gene. Bootstrap analysis (1000 replications) was used to assess significance of the branches. The amino acid sequence of the *HA1* domain was determined for further analysis of the mutations.

## Results

### Epidemiological analysis

**Fig. 1:**
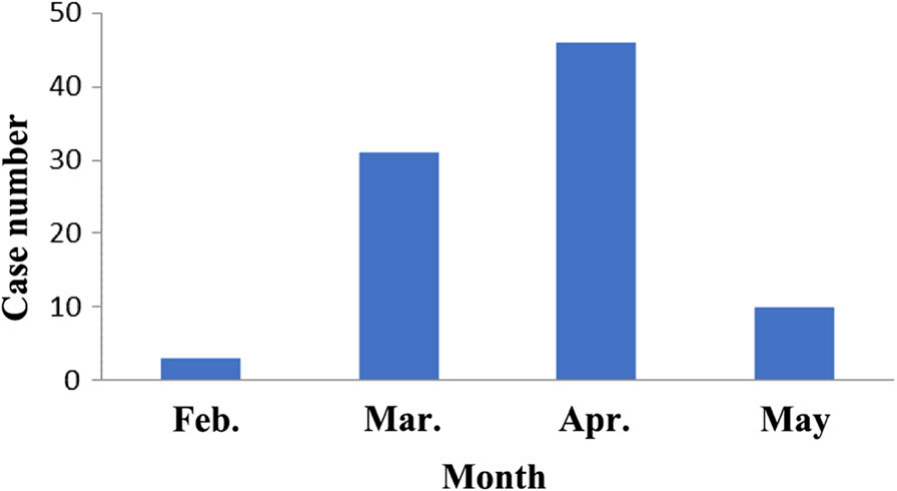
Monthly distribution of influenza B cases in Guangzhou City during 2016

From January 2016 to May 2016, we recruited 90 consecutive patients with influenza B from the First Hospital of Guangzhou Medical University. These patients accounted for 56.25% of all patients infected by the influenza B virus at our institution during that time. Influenza B virus infections occurred as early as February, increased during March, peaked during April, and declined during May (Fig. 1). Eight of the 90 patients also had infections with the influenza A virus. There were 38 males and 52 females, the mean age was 39.8 ± 20.4 years, and the age range was 14 to 74 years-old. Among 72 throat swab specimens, CPE and hemagglutination tests were positive in 53 (73.6%).

### Cladogram analysis of the HA1 domain

**Fig. 2:**
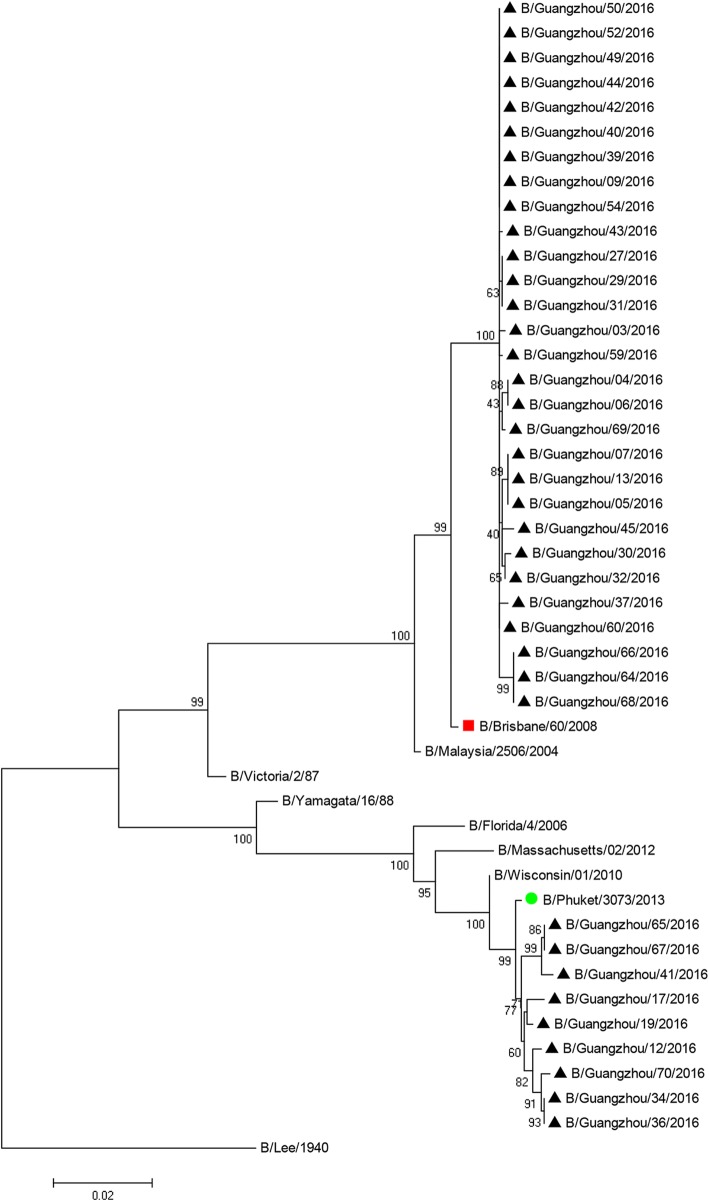
Cladogram of influenza B virus isolates from Guangzhou during 2016, based on the HA1 domain of hemagglutinin

We analyzed the HA1 domain of the *HA* gene in 43 samples to classify the lineages of the isolates that were present during early 2016 in Guangzhou (Fig. 2, Additional file 1: Figure S1). The results show that the Victoria and Yamgata lineages, which have the B/Lee/40 strain as a common ancestor (the “outgroup”), were both prevalent. Among the 43 isolates, 34 (79.1%) were in the Victoria lineage and 9 (20.9%) were in the Yamagata lineage. The 34 isolates in the Victoria lineage were most closely related to the B/Brisbane/60/2008 strain (characterized by WHO in 2009–2011), and more distantly related to B/Malaysia/2506/2004 strain (characterized by WHO in 2006–2008) and the B/Victoria/2/87 strain. The 9 isolates of the Yamagata lineage were most closely related to the B/Phuket/3073/2013 strain (characterized by WHO in 2015–2016), and more distantly related to the B/Wisconsin/1/2010 strain (characterized by WHO in 2009–2011), the B/Massachusetts/2/2012 strain (characterized by WHO in 2013–2015), the B/Florida/4/2006 strain (characterized by WHO in 2006–2008), and the B/Yamagata/16/88 strain.

### Mutations in the HA1 domain

We further analyzed mutations in the HA1 domain in isolates of the Victoria lineage (Table 1, Additional file 2: Figure S2). Relative to B/Brisbane/60/2008, there were 12 mutation sites in the *HA1* domain in our Victoria isolates: I112M (*n* = 3), I132V (*n* = 34), N144D (*n* = 34), A169T (*n* = 3), I190M (*n* = 3), C196S (*n* = 1), E198D (*n* = 1), A214T (*n* = 34), T236I (*n* = 6), I261L (*n* = 1), I261F (*n* = 1), and A291T (*n* = 2). Four of these mutations — I132V, N144D, C196S, and E198D — were in antigenic epitopes.

**Table 1:**
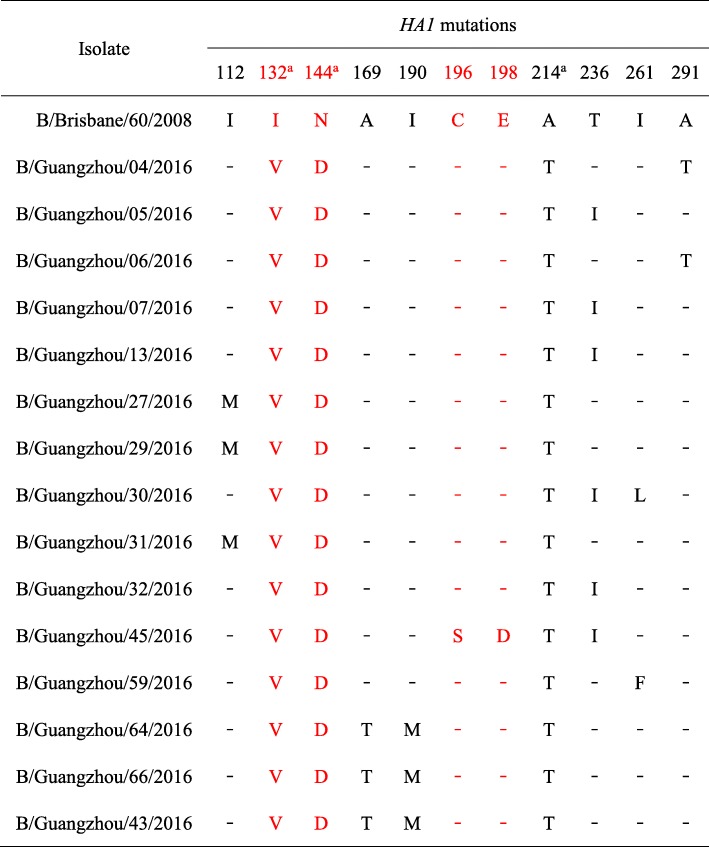
HA1 mutations in influenza B virus isolates from the Victoria lineage

^a^All 34 isolates were mutated at this site

Red font indicates antigenic epitopes

We performed the same analysis in the Yamagata lineage (Table 2, Additional file 2: Figure S2). Relative to B/Phuket/3073/2013, there were 5 mutation sites in the *HA1* domain of our Yamagata isolates: T12 M (*n* = 3), L1871Q (*n* = 9), K226R (*n* = 2), N232D (*n* = 1), and M266 V (*n* = 9). None of these mutations were in antigenic epitopes. The GenBank accession numbers for sequences of isolates collected in the present study are shown in Additional file 3: Table S1.

**Table 2:** HA1 mutations in influenza B virus isolates of the Yamagata lineage

Isolate	*HA1* mutations				
	12	187^a^	226	232	266^a^
B/Phuket/3073/2013	T	L	K	N	M
B/Guangzhou/41/2016	M	Q	–	–	V
B/Guangzhou/65/2016	M	Q	–	–	V
B/Guangzhou/67/2016	M	Q	–	–	V
B/Guangzhou/17/2016	–	Q	R	–	V
B/Guangzhou/19/2016	–	Q	R	–	V
B/Guangzhou/12/2016	–	Q	–	D	V

^a^mutation was observed in all 9 isolates

### Mutation screening for influenza B virus resistance to NA inhibitors

We also analyzed the *NA* gene of 20 samples for 4 specific mutations known to confer resistance to NA inhibitors: G109E, R152K, D198N, and G402S [20, 23, 24] (Additional file 4: Table S2). None of the isolates had mutations responsible for influenza B virus resistance to NA inhibitors.

## Discussion

We analyzed the HA1 domain of the *HA* gene in different isolates of influenza B virus collected from Guangzhou during 2016, and compared it to the WHO-recommended vaccine strain for that period. Eight of the 90 included patients also had influenza A virus infections. Analysis of 43 influenza B viral cultures indicated that 34 isolates were in the Victoria lineage, and 9 were the Yamagata lineage. Our analysis of mutation sites within the HA1 domain of *HA* indicated that some of the isolates in the Victoria lineage, but none of the mutations in the Yamagata lineage, were located in antigenic epitopes.

The vaccine to be used against influenza viruses in China is determined by the WHO. Thus, there must be consistency between the WHO-recommended vaccine strain and the prevalence of different strains in the population for the effective prevention and control of influenza. The WHO provided evidence that the prevalence of influenza during 2016 was greater than in previous years [25]. Our analysis of influenza B virus isolates indicated the Victoria lineage was more prevalent than the Yamagata lineage in our population (76.3% vs. 23.68%). These results are consistent with the results shown for China summarized by the WHO in 2016 [21]. The B/Phuket/3073/2013 vaccine strain recommended by WHO in 2015 to 2016 in the Northern Hemisphere is in the Yamagata lineage, so this vaccine provided no protection against viruses of the Victoria lineage. In contrast, viruses of the Yamagata lineage in Guangzhou were most similar to the B/Phuket/3073/2013 strain (Fig. 2). Therefore, the vaccine that was against influenza B was protective against only 24% of the influenza viruses in our participants. Notably, similar surveillance analyses of circulating influenza B virus during 2016 in other regions, including Thailand [26], showed that the Yamagata strains were predominant, thus confirming the presence of locoregional variations in influenza B virus infections.

The partial mismatch between the circulating strains and vaccine strains of influenza B viruses in the present study is similar to that reported for Guangzhou during 2009 [9]. In addition, a similar characterization of influenza viruses in east-central China during the same time (2009 to 2010) indicated that all 5 isolates analyzed were closely related to the vaccine strain [10]. This highlights the importance of influenza B virus monitoring for different regions. The proximity of Guangzhou to the tropics, where the prevalence of different influenza B strains differ, should be considered in the development of future vaccines for this region of China.

Vaccination is the most effective strategy for prevention of influenza. The traditional vaccine against influenza is trivalent (although there has been broad acceptance of the tetravalent vaccine in North America and elsewhere), and contains two seasonal influenza A virus strains, and one influenza B virus strain (either Victoria or Yamagata). Thus, this trivalent vaccine may offer little protection against influenza B when both lineages are simultaneously present in a population, as in the present study and previous studies [27, 28]. Mismatch of the vaccine with the circulating strains may occur, as observed in the present study, and in previous studies. For example, Moa et al. [29] reported a one-third mismatch in Australia from 2001 to 2014. The WHO has recently recommended a quadrivalent vaccine, with the addition of one more strain of the influenza B virus. In particular, the quadrivalent vaccine recommended by WHO for 2015 to 2016 contained a B/Brisbane/60/2008-like strain, which was prevalent in Guangzhou. During February 2012, the U.S. Food and Drug Administration (FDA) approved the first quadrivalent vaccine that contained influenza B viruses of the Victoria and Yamagata lineages. Other studies have shown similar immunogenicity of the quadrivalent and trivalent vaccines, with comparable safety [30]. Thus, the quadrivalent vaccine may offer greater protection for more geographical regions, especially when the Victoria and Yamagata lineages of the influenza B virus are present.

Influenza B viruses evolve more slowly than influenza A viruses [31]. However, evidence shows that viruses of the Victoria and Yamagata lineages have persistent gene mutations, and that the influenza B virus also undergoes antigen switching [31–33]. Although there are variations of antigenicity in the influenza viruses, not all mutations are epidemiologically important.

The HA1 domain has four main antigenic epitopes: 120-loop (116–137), 150-loop (141–150), 160-loop (162–167), and 190 spiral structure (194–202) [34, 35]. Moreover, recent research has suggested the presence of additional antigenic residues beyond these known regions [36]. Thus, changes in the amino acids of these regions may alter viral antigenicity, and potentially change the direction of viral evolution as new lineages evolve [35]. In this study, we found some antigenicity-related variations in the HA1 domain of influenza B viruses in the Victoria lineage, including I132V, N144D, C196S, and E198D. Among these variations, C196S and E198D were only in a single isolate, but all isolates had the I132V andN144D variations (120-loop and 150-loop).

To qualify as a new clade, a lineage must have the substitution of at least 4 amino acids in 2 to 3 antigenic epitopes in the *HA1* domain [37]. Among the isolates in our study, 15 met these criteria, and 1 (B/Guangzhou/45/2016) had the substitution of 6 amino acids in 4 antigenic epitopes. Thus, according to these criteria, there were changes in the antigenicity of viruses in the Victoria lineage in Guangzhou. Although we did not examine a large number of isolates, our data indicate the prevalence of mutated viruses in the Victoria lineage appears to be high in the general population. According to WHO statistics, infection with the influenza B virus was a regional pandemic during 2016, as in 2010 and 2012, and viruses of the Victoria lineage were predominant (72%) [21].

The present study is limited due to its descriptive nature and small sample size. Furthermore, the data were obtained from a single medical center, and for only part of 2016. Therefore, we may have missed seasonal patterns of influenza B virus infections, which are known to occur in southern China [8]. In addition, we focused on the *HA* gene, because our examination of the *NA* gene indicated no mutations in regions that confer resistance to NA inhibitors. Another limitation is that we had no direct neutralization testing data, and only examined mutations in previously described antigenic sites. Additional multicenter studies with larger cohorts are necessary to confirm the results reported here.

## Conclusion

Our results indicate that most influenza B virus strains in Guangzhou during 2016 were in the Victoria lineage, suggesting there were likely to have alteration in viral antigenicity. Moreover, the influenza B vaccine strain recommended by WHO for 2015 to 2016 in the Northern Hemisphere provided little protection against the influenza B virus isolates in Guangzhou during 2016, most of which were in the Yamagata lineage. This suggests selection of the influenza vaccine strain for use in Guangzhou and elsewhere in China should consider regional conditions, rather than worldwide conditions. Furthermore, a quadrivalent vaccine that protects against viruses of both the Victoria and Yamagata lineages may provide better protection than the traditional trivalent vaccine. The rapid evolution of the influenza B virus and selection pressures by the immune system may lead to the continuing evolution of new mutations that escape immune surveillance. Thus, continuous monitoring of influenza isolates may provide earlier identification of strains with antigenic variations, and this will be helpful for the accurate prediction of vaccine efficacy.

## Additional files

Additional file 1: **Figure S1.** Nucleotide sequences of HA1 domain from the influenza B virus isolates in this study. (TXT 81 kb)

Additional file 2: **Figure S2.** Sites of HA1 mutations in the influenza B virus isolates in this study, relative to the reference strains for the Victoria lineage (B/Brisbane/60/2008) and the Yamagata lineage (B/PHUKET/3073/2013_BA). (DOC 1800 kb)

Additional file 3: **Table S1.** GenBank accession numbers for sequences of isolates reported in the present study (DOCX 15 kb)

Additional file 4: **Table S2.** Screening for *NA* mutations in influenza B virus isolates at sites known to confer resistance to NA inhibitors (DOCX 15 kb)

## Abbreviations

CPE: Cytopathic effect
FBS: Fetal bovine serum
*HA*: *Hemagglutinin A*
NA: Neuraminidase
ORF: Open reading frame
RT-PCR: Reverse transcription-polymerase chain reaction
