# Supplementary figures and images for: Analysis of influenza B virus lineages and the HA1 domain of its hemagglutinin gene in Guangzhou, southern China, during 2016
Source: Virol J. 2018 Nov 14;15:175. doi: 10.1186/s12985-018-1085-5 (PMC6236879; doi:10.1186/s12985-018-1085-5)

1. Victoria


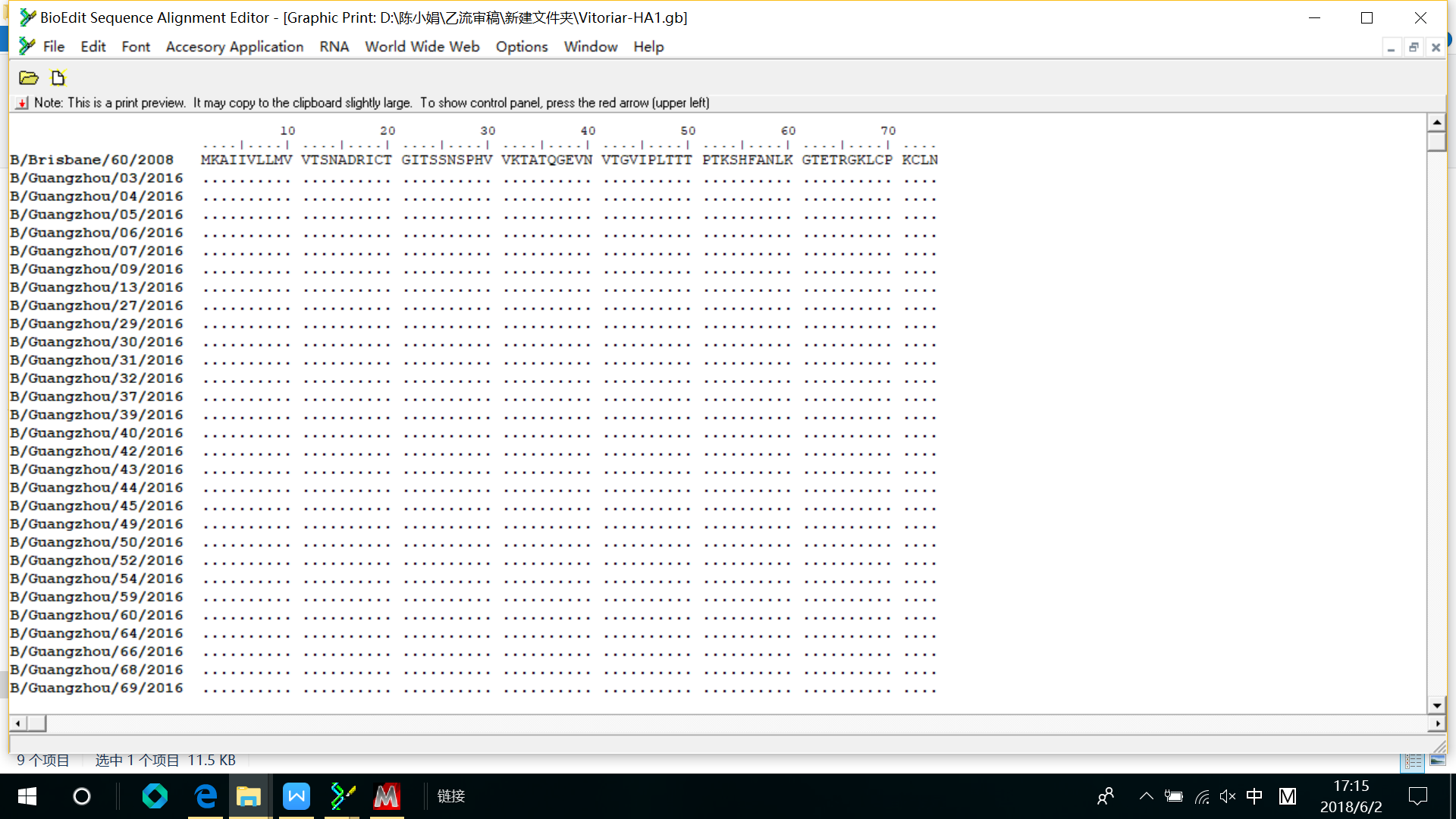


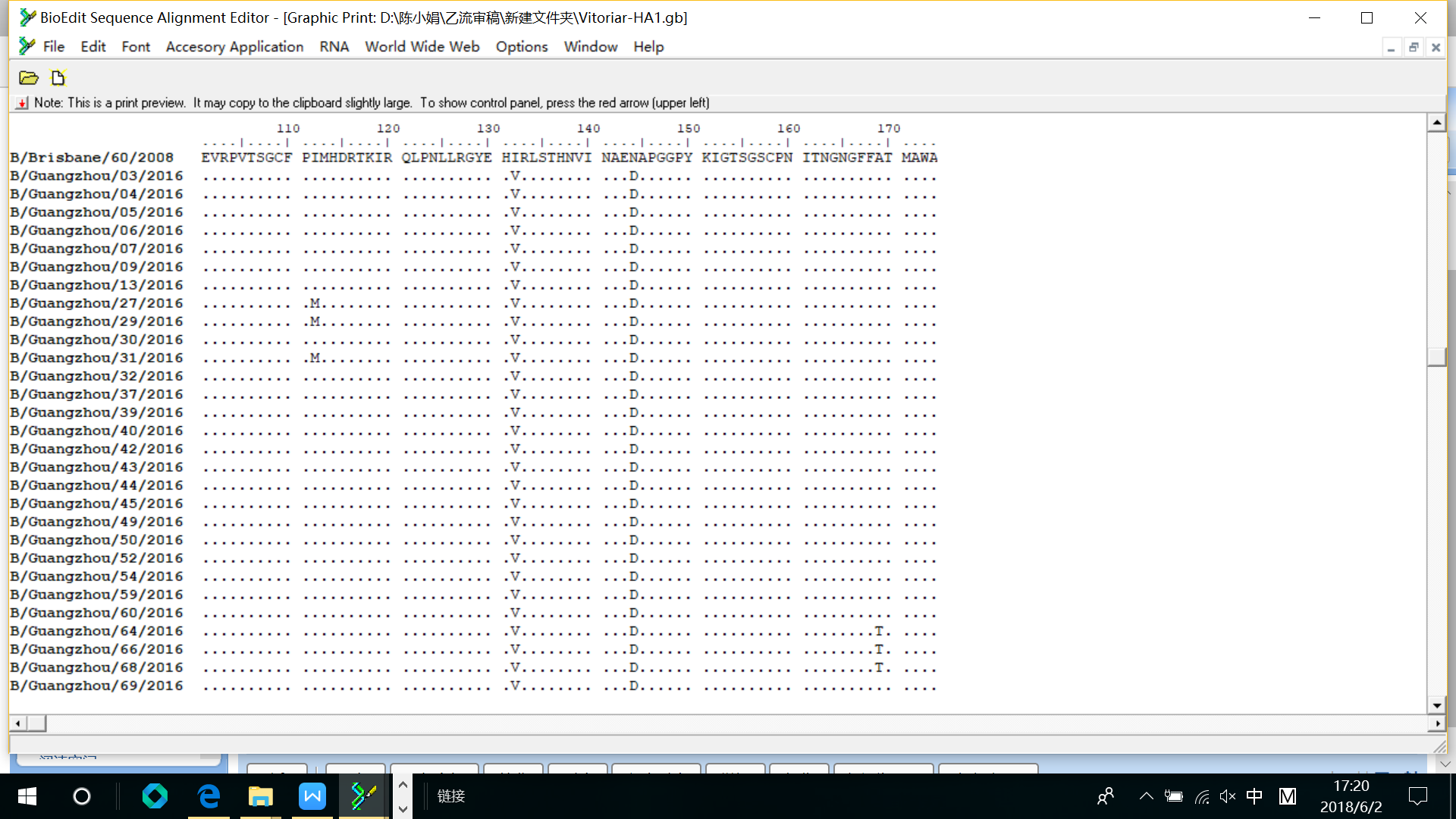


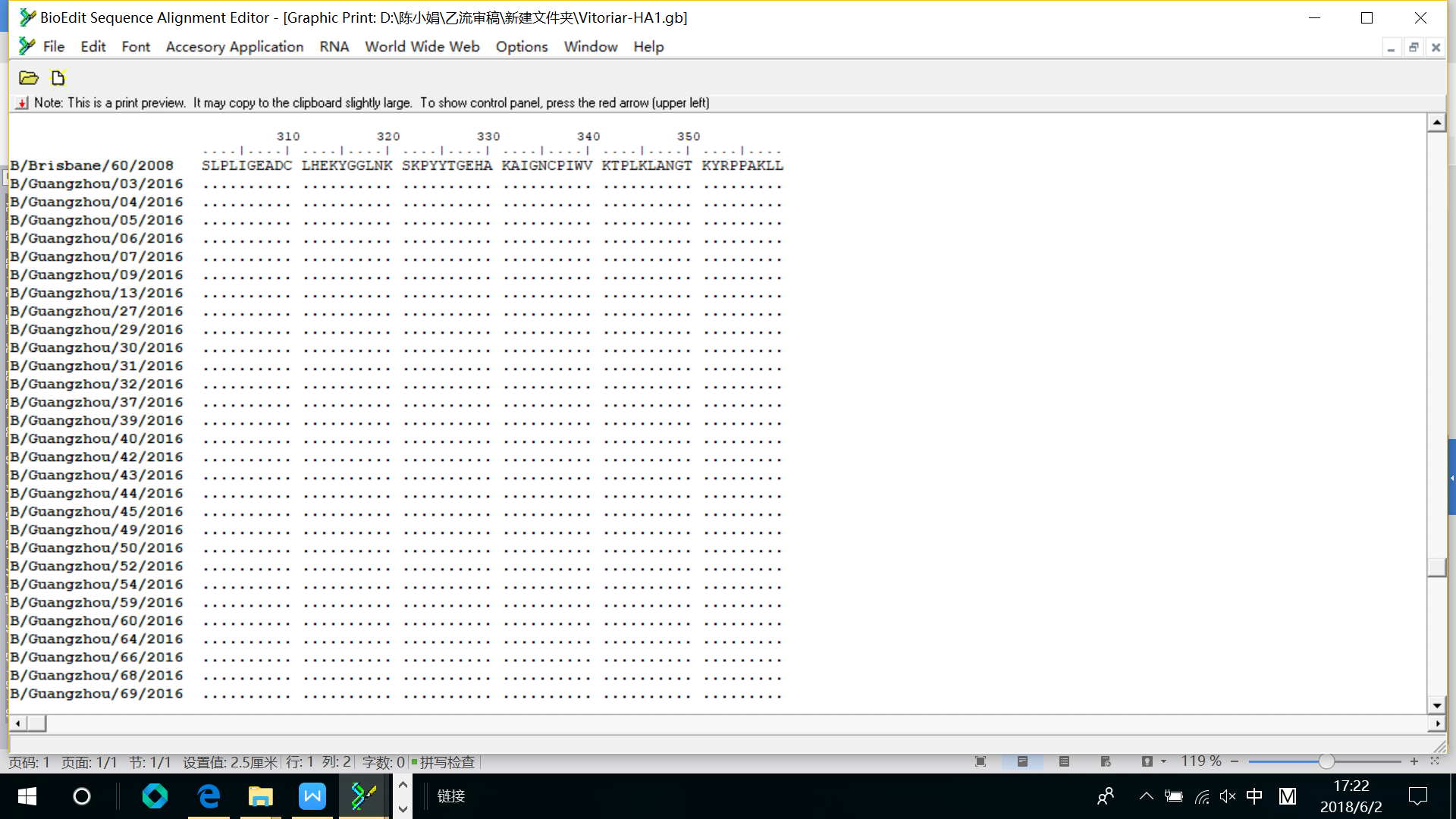


1. Yamagata


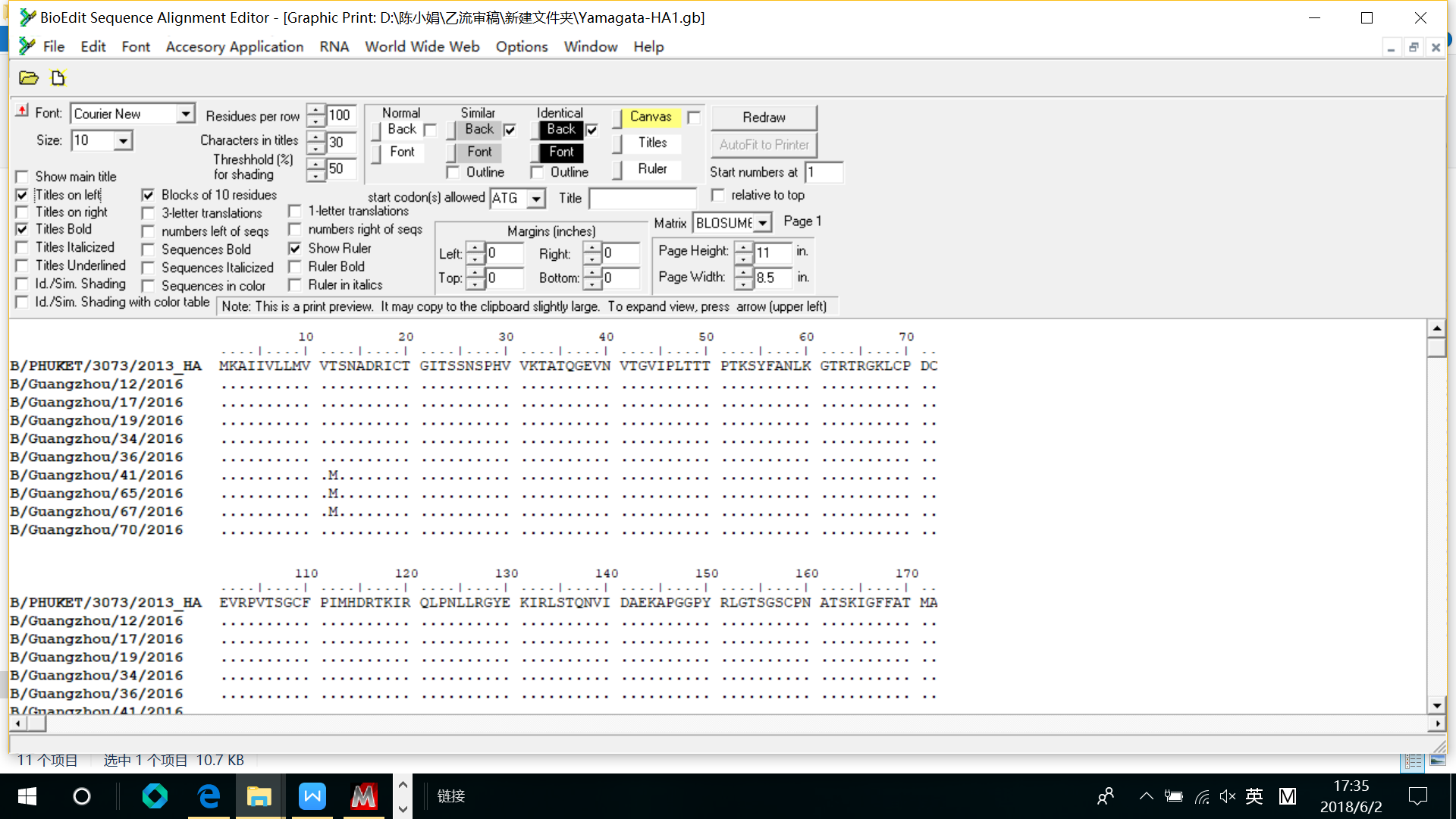


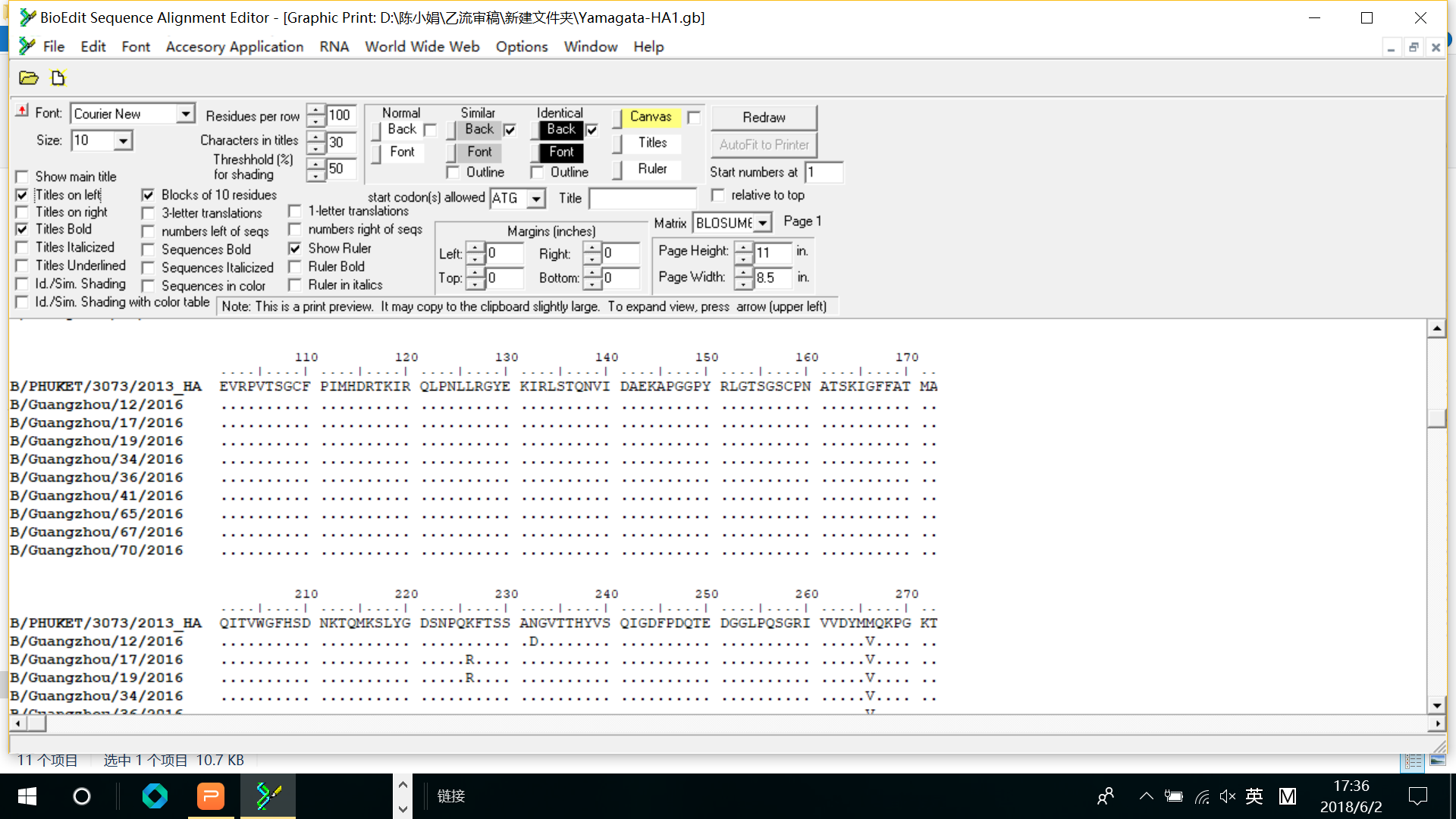


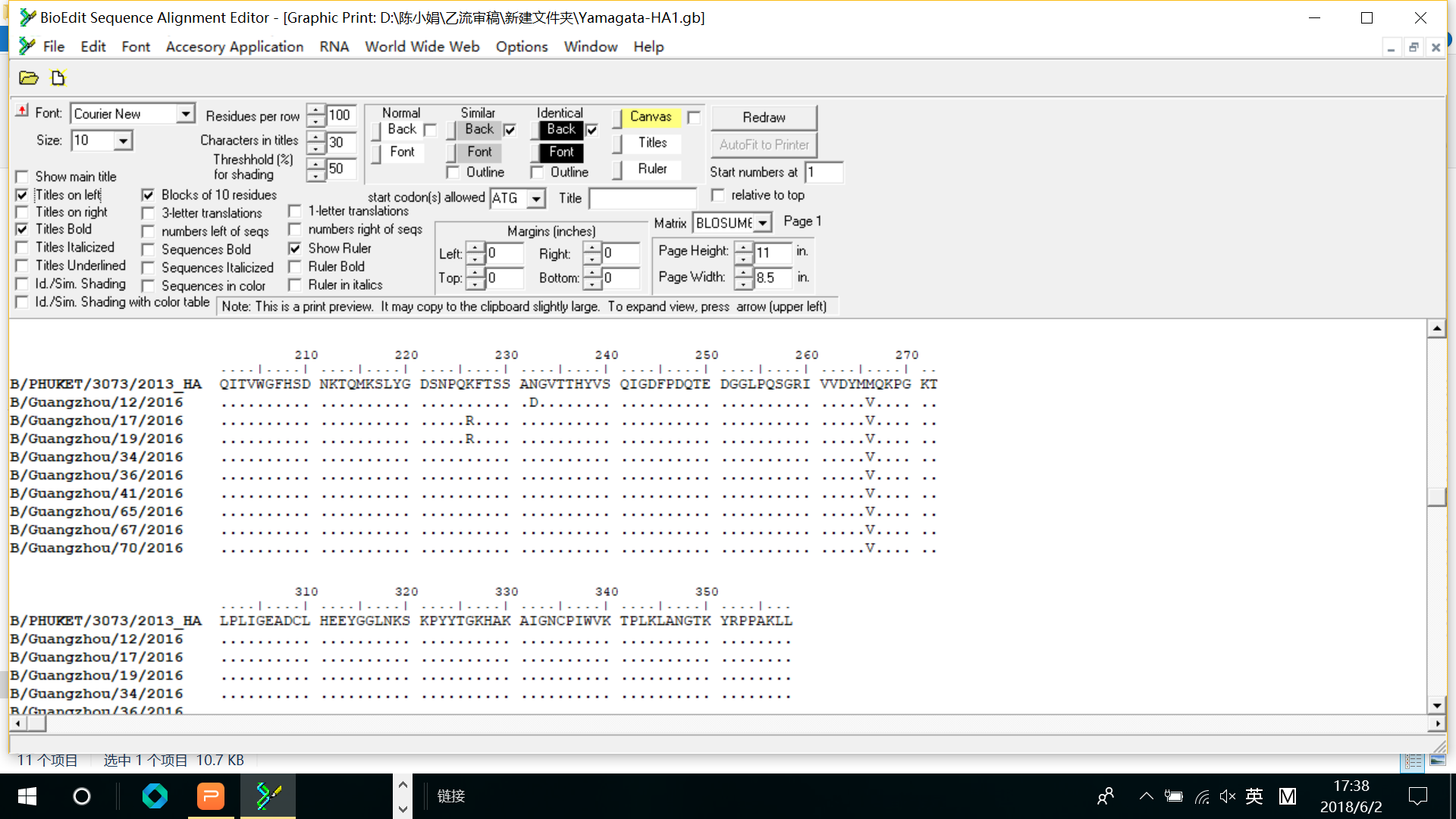


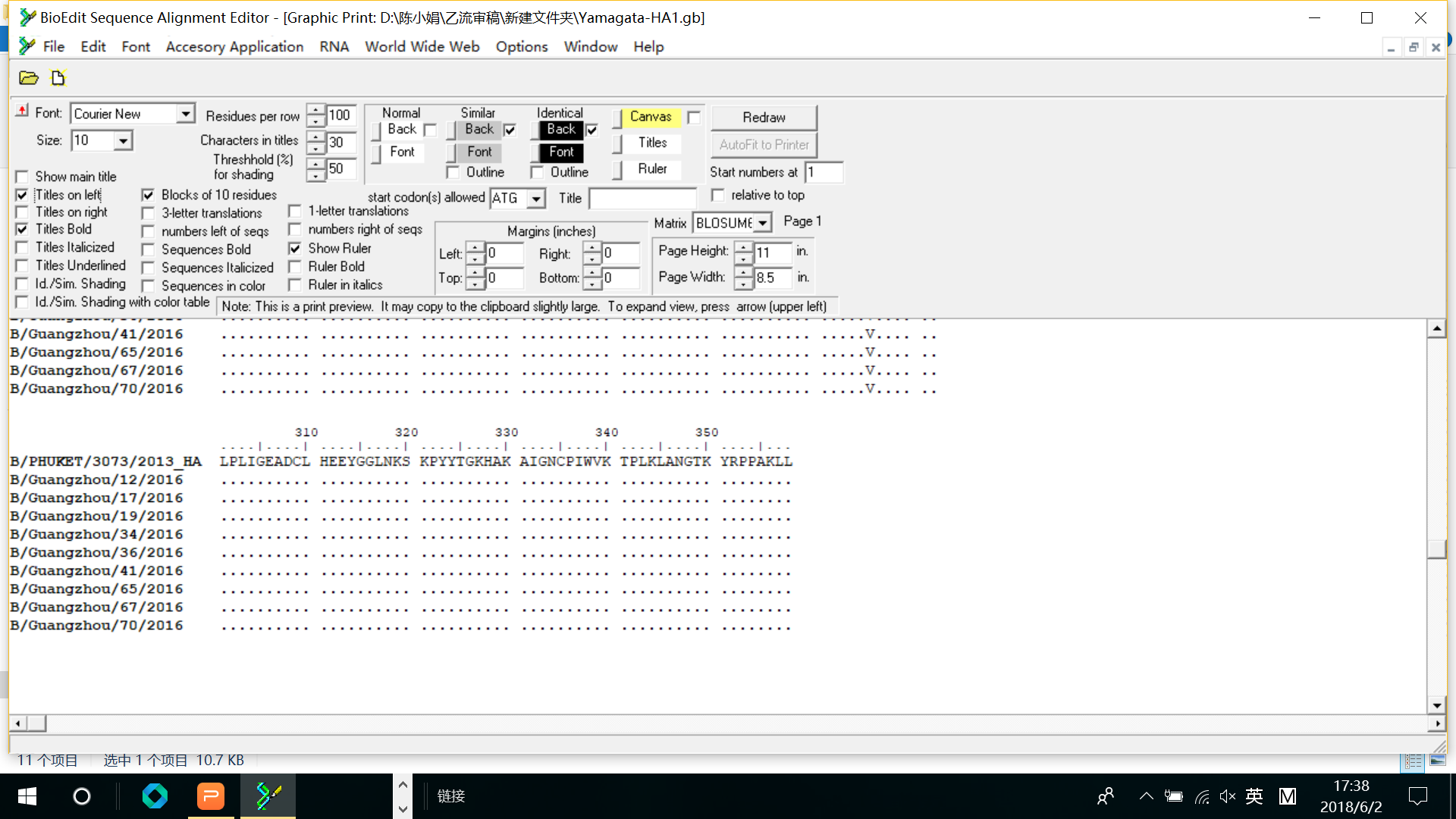

Supplement: Supplementary file 2 — Figure S2. Sites of HA1 mutations in the influenza B virus isolates in this study, relative to the reference strains for the Victoria lineage (B/Brisbane/60/2008) and the Yamagata lineage (B/PHUKET/3073/2013_BA). (DOC 1800 kb) [file 12985_2018_1085_MOESM2_ESM.doc]
