# Supplementary material for: Analysis of influenza B virus lineages and the HA1 domain of its hemagglutinin gene in Guangzhou, southern China, during 2016
Source: Virol J. 2018 Nov 14;15:175. doi: 10.1186/s12985-018-1085-5 (PMC6236879; doi:10.1186/s12985-018-1085-5)
Supplement: Supplementary file 3 — Table S1. GenBank accession numbers for sequences of isolates reported in the present study (DOCX 15 kb) [file 12985_2018_1085_MOESM3_ESM.docx]

**Table S1. GenBank accession numbers for sequences of isolates reported in the present study.**

| **Isolate** | **Lineage** | **Accession no.** |
| --- | --- | --- |
| B/Guangzhou/03/2016 | Victoria | KY095068 |
| B/Guangzhou/04/2016 | Victoria | KY095069 |
| B/Guangzhou/05/2016 | Victoria | KY095070 |
| B/Guangzhou/06/2016 | Victoria | KY095071 |
| B/Guangzhou/07/2016 | Victoria | KY095072 |
| B/Guangzhou/09/2016 | Victoria | KY095073 |
| B/Guangzhou/13/2016 | Victoria | KY095075 |
| B/Guangzhou/27/2016 | Victoria | KY095078 |
| B/Guangzhou/29/2016 | Victoria | KY095079 |
| B/Guangzhou/30/2016 | Victoria | KY095080 |
| B/Guangzhou/31/2016 | Victoria | KY095081 |
| B/Guangzhou/32/2016 | Victoria | KY095082 |
| B/Guangzhou/37/2016 | Victoria | KY095085 |
| B/Guangzhou/39/2016 | Victoria | KY095086 |
| B/Guangzhou/40/2016 | Victoria | KY095087 |
| B/Guangzhou/42/2016 | Victoria | KY095089 |
| B/Guangzhou/43/2016 | Victoria | KY095090 |
| B/Guangzhou/44/2016 | Victoria | KY095091 |
| B/Guangzhou/45/2016 | Victoria | KY095092 |
| B/Guangzhou/49/2016 | Victoria | KY095093 |
| B/Guangzhou/50/2016 | Victoria | KY095094 |
| B/Guangzhou/52/2016 | Victoria | KY095095 |
| B/Guangzhou/54/2016 | Victoria | KY095096 |
| B/Guangzhou/59/2016 | Victoria | KY095097 |
| B/Guangzhou/60/2016 | Victoria | KY095098 |
| B/Guangzhou/64/2016 | Victoria | KY095099 |
| B/Guangzhou/66/2016 | Victoria | KY095101 |
| B/Guangzhou/68/2016 | Victoria | KY095103 |
| B/Guangzhou/69/2016 | Victoria | KY095104 |
| B/Guangzhou/56/2016 | Victoria | KY563922 |
| B/Guangzhou/57/2016 | Victoria | KY563923 |
| B/Guangzhou/58/2016 | Victoria | KY563924 |
| B/Guangzhou/62/2016 | Victoria | KY563925 |
| B/Guangzhou/73/2016 | Victoria | KY563926 |
| B/Guangzhou/12/2016 | Yamagata | KY095074 |
| B/Guangzhou/17/2016 | Yamagata | KY095076 |
| B/Guangzhou/19/2016 | Yamagata | KY095077 |
| B/Guangzhou/34/2016 | Yamagata | KY095083 |
| B/Guangzhou/36/2016 | Yamagata | KY095084 |
| B/Guangzhou/41/2016 | Yamagata | KY095088 |
| B/Guangzhou/65/2016 | Yamagata | KY095100 |
| B/Guangzhou/67/2016 | Yamagata | KY095102 |
| B/Guangzhou/70/2016 | Yamagata | KY095105 |
