# Supplementary material for: Analysis of influenza B virus lineages and the HA1 domain of its hemagglutinin gene in Guangzhou, southern China, during 2016
Source: Virol J. 2018 Nov 14;15:175. doi: 10.1186/s12985-018-1085-5 (PMC6236879; doi:10.1186/s12985-018-1085-5)
Supplement: Supplementary file 4 — Table S2. Screening for NA mutations in influenza B virus isolates at sites known to confer resistance to NA inhibitors (DOCX 15 kb) [file 12985_2018_1085_MOESM4_ESM.docx]

**Table S2. Screening for *NA* mutations in influenza B virus isolates at sites known to confer resistance to NA inhibitors.**

| Isolate | Site of neuraminidase gene | | | |
| --- | --- | --- | --- | --- |
|  | G109E | R152K | D198N | G402S |
| B/Guangzhou/01/2016 | G | R | D | G |
| B/Guangzhou/05/2016 | G | R | D | G |
| B/Guangzhou/07/2016 | G | R | D | G |
| B/Guangzhou/09/2016 | G | R | D | G |
| B/Guangzhou/13/2016 | G | R | D | G |
| B/Guangzhou/17/2016 | G | R | D | G |
| B/Guangzhou/19/2016 | G | R | D | G |
| B/Guangzhou/30/2016 | G | R | D | G |
| B/Guangzhou/32/2016 | G | R | D | G |
| B/Guangzhou/34/2016 | G | R | D | G |
| B/Guangzhou/36/2016 | G | R | D | G |
| B/Guangzhou/37/2016 | G | R | D | G |
| B/Guangzhou/39/2016 | G | R | D | G |
| B/Guangzhou/40/2016 | G | R | D | G |
| B/Guangzhou/41/2016 | G | R | D | G |
| B/Guangzhou/49/2016 | G | R | D | G |
| B/Guangzhou/52/2016 | G | R | D | G |
| B/Guangzhou/56/2016 | G | R | D | G |
| B/Guangzhou/57/2016 | G | R | D | G |
| B/Guangzhou/70/2016 | G | R | D | G |
